# Effect of sclerostin-neutralising antibody on periarticular and systemic bone in a murine model of rheumatoid arthritis: a microCT study

**DOI:** 10.1186/ar4305

**Published:** 2013-09-18

**Authors:** Massimo Marenzana, Alex Vugler, Adrian Moore, Martyn Robinson

**Affiliations:** 1 UCB Pharma, 208 Bath Road, Slough, Berkshire, SL1 3WE, UK; 2 Department of Bioengineering, Imperial College London, South Kensington Campus, London SW7 2AZ, UK

**Keywords:** Sclerostin, sclerostin antibody, inflammation, collagen-induced arthritis, rheumatoid arthritis, bone loss, focal erosion, micro computed tomography, microCT

## Abstract

**Introduction:**

Patients with chronic inflammatory diseases have increased bone loss and bone fragility and are at increased risk of fracture. Although anti-resorptive drugs are effective in blocking inflammation-induced bone loss, they are less effective at rebuilding bone. We have previously shown that treatment with sclerostin antibody (Scl-AbI) builds bone and can prevent or restore bone loss in a murine model of inflammatory bowel disease. In this study, we tested the effect of Scl-AbI in a murine model of rheumatoid arthritis (the collagen-induced arthritis model, CIA). We hypothesised that sclerostin blockade can protect and restore bone both locally and systemically without affecting progression of inflammation.

**Methods:**

CIA was induced in male DBA/1 mice, which were treated with either PBS or Scl-AbI (10 mg/kg, weekly) prophylactically for 55 days or therapeutically for 21 days (starting 14 days post onset of arthritis). Systemic inflammation was assessed by measuring the serum concentration of anti-CII IgG1, IgG2a and IgG2b by ELISA. Changes in bone mass and structure, either at sites remote from the joints or at periarticular sites, were measured using DEXA and microCT. Bone focal erosion was assessed in microCT scans of ankle and knee joints.

**Results:**

Circulating anti-CII immunoglobulins were significantly elevated in mice with CIA and there were no significant differences in the levels of anti-CII immunoglobulins in mice treated with PBS or Scl-ABI. Prophylactic Scl-AbI treatment prevented the decrease in whole body bone mineral density (BMD) and in the bone volume fraction at axial (vertebral body) and appendicular (tibial proximal metaphysis trabecular and mid-diaphysis cortical bone) sites seen in PBS-treated CIA mice, but did not prevent the formation of focal bone erosions on the periarticular bone in the knee and ankle joints. In the therapeutic study, Scl-AbI restored BMD and bone volume fraction at all assessed sites but was unable to repair focal erosions.

**Conclusions:**

Sclerostin blockade prevented or reversed the decrease in axial and appendicular bone mass in the murine model of rheumatoid arthritis, but did not affect systemic inflammation and was unable to prevent or repair local focal erosion.

## Introduction

Patients with chronic inflammatory diseases - for example rheumatoid arthritis (RA), systemic lupus erythematosus, inflammatory bowel disease (IBD), celiac disease, cystic fibrosis and chronic obstructive pulmonary disease - have increased bone fragility and are at an increased risk of sustaining a bone fracture [1]. Possible reasons for the increased fracture risk include: poor nutritional status, hypovitaminosis D, a decrease in calcium intake, corticosteroids treatment, reduced mobility and exercise, and systemic chronic inflammation. Chronic inflammation is believed to be one of the key factors and is active from the early stages of all of the aforementioned inflammatory diseases [2]. Inflammatory bone loss is regulated by various mediators of the immune system such as the pro-inflammatory cytokines tumour necrosis factor-alpha (TNF-alpha), interleukin-1 beta (IL-1 beta), interleukin-6 (IL-6), or interferon-gamma, which have all been shown to modulate osteoclastogenesis [1]. Other cytokines such as receptor activator of nuclear factor kappa B (RANK), its ligand, RANKL, and osteoprotegerin (OPG) are also critically involved in the pathophysiology of inflammatory bone loss [3]. Moreover, inflammatory factors such as TNF-alpha have also been shown to reduce osteoblast activity, thereby inhibiting the formation of new bone. Although anti-resorptive approaches, such as bisphosphonates, denosumab, IL-1 receptor antagonist, and TNF-alpha antibody have been effective in slowing or blocking inflammation-induced bone loss, they have shown a limited capacity to restore lost bone [2].

RA is an autoimmune disease with both articular and extra-articular involvement. The main consequence of RA is the induction of structural joint damage, a source of physical and social handicap leading to a huge economic cost. Thus, new treatments to stop RA evolution are an important need for both patients and society. In early RA, radiographic demineralisation appears around inflamed joints, while bone erosions appear later [4].

Several Wnt family members seem to be involved in the modulation of the inflammatory response during RA [5-7]. Wnt-7b was shown to be upregulated in the cartilage of osteoarthritic patients and in the synovium of RA patients, where it has been implicated in the production of the pro-inflammatory cytokines TNF-alpha and IL-1-beta [8]. Additional data supporting a role of the Wnt signalling pathway in the pathogenesis of RA comes from the fact that the blockade of Wnt-5a/Frizzled5 signalling decreases IL-6 and IL-15 production during the activation of fibroblast-like synoviocytes (FLSs) [9]. Moreover, Wnt-1 was shown to regulate fibronectin and promote matrix metalloproteinase-3 (MMP-3) production in FLSs [10] and Wnt-1-inducible signalling pathway protein-2 (WISP2) was shown to be upregulated in RA synovial fibroblasts [11]. Therefore, as well as direct effects on bone formation, Wnt family members might also have an indirect impact on bone metabolism by modulating inflammatory responses. Inflammation can also affect the regulation of Wnt signaling [5]. The induction of Dickkopf-1 (DKK1) by TNF-alpha and the consequent inhibition of the Wnt signalling pathway have been observed both in human RA [12,13] and in murine arthritis models [14]. In this case, TNF-alpha-mediated DKK1 regulation represents a major factor in the blockade of osteoblast development, resulting in the inflamed joint going unrepaired [15]. Therapeutic scavenging of DKK1 by inhibitory antibodies in the human TNF transgenic mouse model resulted in an increase in bone formation, leading to the maintenance of joint integrity as well as the local development of osteophytes: bony spurs that are characteristic of degenerative joint remodelling processes [16].

Sclerostin blockade with a monoclonal antibody (Scl-AbI) has been shown to stimulate bone formation in both humans and animal models [17]. We have recently shown that systemic bone loss in a mouse model of IBD was prevented and reversed with a blocking antibody to sclerostin [18]. This caused reactivation of Wnt signalling in osteoblasts, which was reflected in a reduction of the circulating osteoclastic marker (tartrate-resistant acid phosphatase, TRAP) and an increase of the osteoblastic marker (osteocalcin).

Given the prominent role of Wnt signaling in RA, we hypothesised that sclerostin blockade may exert a bone-protective or restoring action, both locally and systemically, without affecting progression of inflammation (consistent with our previous study). To test this hypothesis, we have used a murine model of rheumatoid arthritis, the type II collagen-induced arthritis (CIA) model. This model is known to induce both systemic osteopenia and periarticular bone loss leading to loss of joint integrity, as seen in RA in patients [16,17]. We treated CIA mice with Scl-AbI and focused our analysis on the changes in bone mass and structure either at sites remote from the joints and at periarticular sites using dual-energy X-ray absorptiometry (DEXA) and micro computed tomography (microCT).

## Methods

### Mice

Eight- to ten-week-old male DBA/1 mice were purchased from Harlan Laboratories (Bicester, UK). Mice were housed in cages in an environmentally controlled room (temperature 21°C to 23°C and relative humidity 38% to 50%) on a 12-hour light/dark cycle according to UK Home Office regulations. Animals had access to RM1 food pellets (Lillico, Betchworth, UK) and water *ad libitum*. Animals were acclimatised for four weeks before use.

The work was conducted in the UK according to the Animals Scientific Procedures Act (1986) and was covered by a Project Licence that was subject to both ethical review by AURICC (Animal Use in Research Coordinating Committee) and Government review (UK Home Office).

### Induction/assessment of collagen-induced arthritis and bone mineral density

Collagen-induced arthritis (CIA) was induced as described earlier [21]. In brief, cages of male DBA/1 mice (*n *= 5 per cage) were weighed and placed into groups (*n *= 15 per group) to give similar initial body weights. Chicken type II collagen (CII) (MD Biosciences, Zurich, Switzerland) was dissolved in 0.1 M acetic acid at 2 mg/mL and constantly rolled overnight in the dark at 4°C. The next day (Day 0) the CII solution was emulsified in an equal volume of complete Freund's adjuvant (CFA, Sigma-Aldrich, Gillingham, UK) to give a 1 mg/mL emulsion. DBA/1 mice were injected with 0.1 mL (100 μg CII) of emulsion into the base of the tail by an intradermal route under anaesthesia. Fourteen days post-sensitisation mice were given a booster injection of CII, by intradermal route as above, but with incomplete Freund's adjuvant (Sigma-Aldrich), instead of CFA. Mice were assessed for signs of arthritis from day 14 onwards. When signs of arthritis were seen, mice were weighed and clinically scored. Clinical score was graded on a 0 to 3 scale as follows: grade 0, no swelling; grade 1, wrist or ankle swollen; grade 2, wrist/ankle and pad swollen; grade 3, wrist/ankle, pad and digits swollen. Clinical score was the sum of all four paws (maximum score per mouse was 12).

### Study design

In the prophylactic study, arthritis was induced in two groups of male DBA/1 mice (*n *= 15/group). Starting from one day prior to collagen II sensitisation, one group of mice was treated with 10 mg/kg (100 μL subcutaneously once per week) of a monoclonal antibody directed against sclerostin [18-22] and the other with PBS (100 μL subcutaneously once a week). A third group of mice (*n *= 6) was used as age-matched, healthy, untreated controls. The prophylactic study was terminated 55 days post-sensitisation.

In the therapeutic study, arthritis was induced in three groups of male DBA/1 mice. For each animal, onset of arthritis was considered to be day 1. One group of mice was treated with 10 mg/kg of Scl-AbI (*n *= 11) and a second group with PBS (100 μl subcutaneously once a week) (*n *= 14) starting from 14 days post-onset of arthritis. These groups were terminated 21 days after starting treatment. Arthritis was induced in a third group of mice, with each animal killed 14 days after onset of arthritis (*n *= 7) to act as a baseline for treatment comparisons. A fourth group of mice (*n *= 4) was used as age-matched, healthy, untreated controls.

### Bone assessment and analysis

At termination of the studies, mice were killed and total (excluding the skull) and vertebral bone mineral density (BMD) were assessed using dual-energy X-ray absorptiometry (DEXA, Lunar PIXImus, GE Medical Systems, Little Chalfont, UK). Dissected hind limbs (from the hip joint to the ankle) and L5 lumbar vertebrae were scanned by microCT (SCANCO, Brüttisellen, Switzerland, μCT35) at 70 kVp and 160 μA, with resolution of 6 μm isotropic voxel size. Segmentation, selection of regions of interest and computation of three-dimensional microstructural parameters were performed by SCANCO proprietary software (image processing language, IPL), as previously reported [23]. Noise was removed from images using a Gaussian filter (sigma = 0.8 and support = 1) and fixed threshold of 500 mg/cm^3 ^was used to segment bone from the background. Analysis of the tibia was performed in two regions: 1) the mid-diaphysis cortical bone, a 0.6 mm-long segment (or 100 tomograms) centred over the tibial midline, and 2) the proximal tibial metaphysis, a region starting 0.30 mm from an anatomic landmark in the growth plate and extending 1.2 mm (or 200 tomograms) distally. The vertebral bodies of L5 vertebra, including upper and lower discs, were selected from the scout view images of the whole mouse spine. Regions of interest were manually drawn to include trabecular bone and exclude cortical bone in either the tibial metaphysis or the L5 vertebral body. To evaluate bone focal erosion in the ankle joint and knee joint, these anatomical regions were selected by manually drawn regions of interest (640 tomograms centered over the synovial space for the knee joint and 700 tomograms including all the bones in the tarsus and the calcaneus). Bone volume (BV) and bone surfaces (BS) of these entire articular joints were then computed. To evaluate the surface density of the periarticular bone (indicating focal erosion on the bone surfaces) the ratio BS/BV was used similarly as previously reported [23]. However, since BS/BV depends on bone volume changes, another independent parameter was introduced to estimate the smoothness of the periarticular bone surfaces. This was obtained by first recalculating the parameter BS - which was re-named BSsmooth - after applying a strong smoothing filter (Gaussian with sigma = 2.5 and support = 5) to the microCT images. Then the ratio of BSsmooth over the original BS was computed as estimator of surface smoothness. This estimator has a value close to one for perfectly smooth surfaces where additional smoothing does not cause changes in the parameter BS, and close to zero for extremely rough surfaces where strong smoothing dramatically alters the value of BS.

### Serological analysis of immunoglobulins

The presence of anti-CII immunoglobulins (IgG1, IgG2a and IgG2b) was determined by ELISA in serum samples obtained at termination of the prophylactic study (Day 55). In brief, 96-well ELISA plates were coated with 5 μg/mL CII (MD Biosciences) dissolved in PBS (100 μL per well). Plates were covered and stored overnight at 4°C. The next day plates were washed twice with PBS on an automated plate washer then blocked with 1% bovine serum albumin (BSA) in PBS (200 μL per well) and placed on a plate shaker for 30 minutes at room temperature. Plates were then washed twice with PBS. Serum samples were diluted in PBS at 1 in 10,000 for detection of anti-CII IgG1 and IgG2a and 1 in 20,000 for detection of anti-CII IgG2b, 100 μL of each sample were plated out in duplicate. Plates were placed on a plate shaker for 1 hour at room temperature and then washed twice with PBS. Anti-mouse IgG1, IgG2a and IgG2b horseradish peroxidase-conjugated antibodies (AbD Serotec, Kidlington, UK) were diluted from stock 1 in 5,000 in PBS containing 1% BSA and 100 μL per well added to plates. Plates were then placed on a plate shaker for 1 hour at room temperature and then washed four times with PBS. 3, 3', 5, 5' tetramethylbenzidine substrate (Sigma-Aldrich) was added to the plates (100 μL per well). The reaction was stopped after 10 minutes with 100 μL 1N sulphuric acid. Transmitted light absorbance was read on a plate reader at 450 nm wavelength.

### Statistics

Data were analysed using GraphPad Prism v.5.0. (GraphPad Software, La Jolla, CA, USA) Data are represented as means +/- standard deviations with the exception of the clinical scores and serological results, which are represented as means +/- standard error of the mean. Statistical analysis of all data, except for the clinical scores, was conducted by one-way ANOVA with Tukey's post hoc test. Differences between clinical score were analysed by Mann-Whitney test. Values of *P *< 0.05 were considered statistically significant.

## Results

### Prophylactic study - treatment with Scl-AbI has no significant effect on clinical scores and systemic inflammation in CIA mice

**Figure 1:**
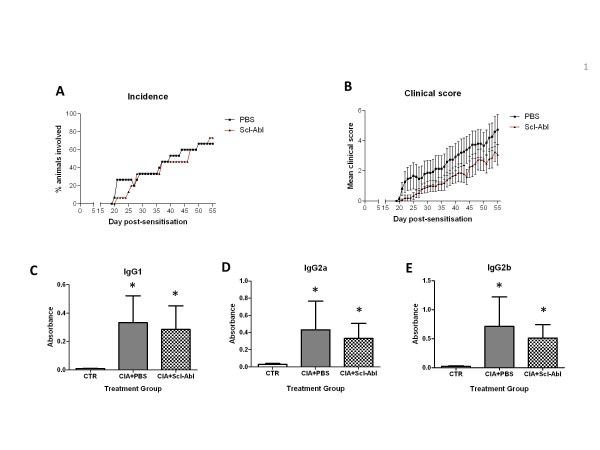
**Prophylactic administration of sclerostin antibody to CIA mice has no effect on clinical scores and systemic inflammation**. Two groups of male DBA/1 mice were sensitized to collagen type II to induce arthritis. One group were treated with vehicle (collagen-induced arthritis (CIA) + PBS; *n *= 15) and the other group received 10 mg/kg sclerostin antibody (Scl-AbI) (CIA + Scl-AbI; *n *= 15). PBS or Scl-AbI were injected subcutaneously once weekly until termination of the experiment (day 55). **(A) **Incidence (reported as percentage of animals involved) and **(B) **clinical score of arthritis were assessed daily and are displayed as mean ± SEM. **(C, D, E) **Bar plots ± SEM represent serum concentrations of anti-CII immunoglobulins IgG1, IgG2a and IgG2b respectively in each group. Serum from age-matched healthy control mice (CTR; *n *= 6) were analysed for baseline levels of anti-CII antibodies. Differences between clinical scores were analysed by Mann-Whitney test. The one-way ANOVA test with Tukey's post hoc comparison was used for statistical evaluation of the serum concentration results shown in the bar plots. *P < 0.05 for the difference versus CTR.

Mice receiving Scl-AbI had an incidence of disease that was not significantly different from mice treated with PBS (Figure 1A). Furthermore, the mean clinical score did not significantly differ in the two groups throughout the course of the experiment (Figure 1B). There was little change in body weight of the mice (approximately 23 g) during the course of the experiment in either the presence or absence of Scl-AbI treatment. Serum samples taken at the end of the study were analysed by ELISA for the presence of circulating anti-CII antibodies. Scl-AbI treatment had no significant effect on the concentration of anti-CII immunoglobulins (IgG1, IgG2a or IgG2b), which were all significantly elevated in PBS-treated arthritic mice compared with healthy controls (Figure 1C, D, E).

### Prophylactic study - treatment with Scl-AbI prevented whole body BMD, axial and tibial bone losses in CIA mice

**Figure 2:**
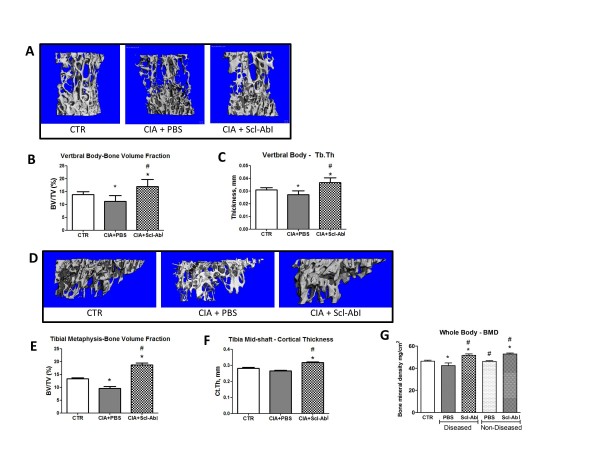
**Prophylactic administration of sclerostin antibody to CIA mice prevents systemic bone loss**. **(A, D) **Representative three-dimensional renditions of the trabecular bone within the L5 vertebral body and the tibial metaphysis, respectively, scanned by micro computed tomography. **(B, C) **Bar plots represent mean (and standard deviation) of the trabecular bone volume fraction (BV/TV) in the vertebral body and the trabecular thickness (Tb.Th). **(E, F) **Bar plots represent mean (and standard deviation) of the trabecular bone volume fraction (BV/TV), the tibial metaphysis and the cortical thickness of the cortical bone in the tibia midshaft. **(G) **Bar plot represents mean (and standard deviation) of the bone mineral density (BMD) assessed by dual-energy X-ray absorptiometry (DEXA). The groups treated with PBS or sclerostin antibody (Scl-AbI) were split into diseased (PBS *n *= 11, Scl-AbI *n *= 11) and non-diseased mice (PBS *n *= 4, Scl-AbI *n *= 4). A group of age-matched healthy controls (CTR *n *= 6) were used to determine baseline bone mineral density (BMD). The one-way ANOVA test with Tukey's post hoc comparison was used for statistical evaluation of all parameters shown in the bar plots. *P < 0.05 for the difference versus CTR; #*P *< 0.05 for the difference versus collagen-induced arthritis (CIA) + PBS (and diseased/non-diseased PBS in graph G).

Mice sensitised to CII, and treated with PBS, showed a statistically significant decrease in the bone volume fraction (BV/TV) in L5 vertebral body (Figure 2A, B) compared with healthy controls, which was primarily due to thinning of the trabeculae (Figure 2C). Prophylactic treatment of the sensitised mice with Scl-AbI prevented these changes and increased both BV/TV and trabecular thickness above untreated healthy controls. Interestingly, similar results were found for the trabecular bone in the tibial metaphysis (Figure 2D, E), despite the relative proximity to the knee joint, which is an area focally targeted in the disease model. The mid-diaphysis cortical bone thickness in the tibia was also reduced in sensitised mice treated with PBS compared to healthy untreated mice, although this trend did not reach statistical significance. Scl-AbI treatment significantly increased tibial cortical thickness compared to both PBS-treated and healthy untreated mice (Figure 2F). This increase in cortical thickness appeared to be due primarily to endosteal bone apposition since the diaphysis total volume (cortical bone volume plus marrow volume) was unchanged between PBS- and Scl-AbI-treated mice, while the marrow volume was significantly decreased (1.6-fold, data not shown).

Mice sensitised to CII showed a statistically significant decrease in whole body BMD as compared to age-matched control mice 55 days post-sensitisation (Figure 2G). Scl-AbI treatment completely prevented this BMD loss in CIA mice and further increased their mean BMD above healthy control level by the end of the experiment (Figure 2G). Whole body BMD within the subpopulations of CII-sensitised mice with either active arthritis (diseased) or those with no signs of arthritis (non-diseased) were assessed. Non-diseased mice had similar total BMD to those of age-matched healthy control mice. However, mice with active disease showed a statistically significant decrease in BMD as compared to non-diseased mice suggesting an association between active inflammation of the paws and loss of whole body BMD (Figure 2G).

Treatment with Scl-AbI also caused a statistically significant increase in total BMD in both diseased and non-diseased mice as compared to PBS-treated diseased and PBS-treated non-diseased mice respectively (Figure 2G).

### Prophylactic study - treatment with Scl-AbI does not protect from focal bone loss in CIA mice

**Figure 3:**
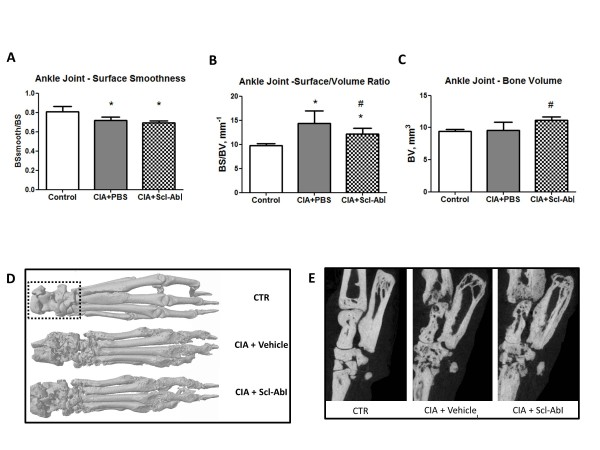
**Prophylactic administration of sclerostin antibody to CIA mice does not prevent focal bone erosion in periarticular bone in the ankle joint**. **(A, B, C) **Bar plots represent mean (and standard deviation of) smoothness parameter (BSsmooth/BS), ratio between bone surface and bone volume (BS/BV), representing surface density, and ankle joint bone volume (BV), respectively. **(D) **Representative three-dimensional renditions of the hind paws scanned by micro computed tomography. **(E) **Representative longitudinal mid-sections on the ankle joint and all the bones in the tarsus. Bone volume and erosion parameters were evaluated in the region of interest displayed on the panel A (dotted selection on control (CTR)). The one-way ANOVA test with Tukey's post hoc comparison was used for statistical evaluation of all parameters shown in the bar plots. *P < 0.05 for the difference versus CTR; #*P *< 0.05 for the difference versus collagen-induced arthritis (CIA) + PBS.

The effect of focal bone erosion on bone microstructure was assessed by microCT imaging of either ankle joint or knee joint. The periarticular bone in the ankles of the mice sensitised to CII showed a statistically significant decrease in the parameter evaluating surface smoothness (BSsmooth/BS, Figure 3A) and increase in the parameter quantifying surface density (BS/BV, Figure 3B) compared to age-matched control mice 55 days post-sensitisation. Treatment with Scl-AbI was able to prevent the changes in the BS/BV but not in the BSsmooth/BS. This effect appeared to be due to an antibody-mediated significant increase in bone volume (Figure 3C), rather than a reduction of the superficial pitting (Figure 3A). This was clearly visualised in the three-dimensional renditions, showing the superficial pitting (Figure 3D) in both the Scl-AbI- and PBS-treated diseased mice. The two-dimensional longitudinal virtual sections of the ankle joint show that bone volume increased internally from the endosteal surfaces (Figure 3E).

**Figure 4:**
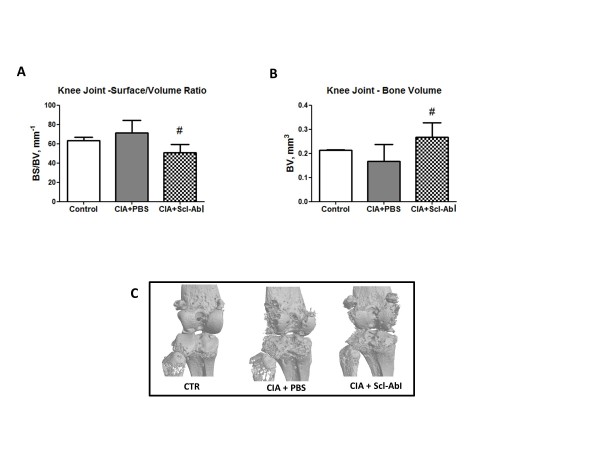
**Prophylactic administration of sclerostin antibody to CIA mice does not prevent focal bone erosion in periarticular bone in the knee joint**. **(A, B) **Bar plots represent mean (and standard deviation of) ratio between bone surface and bone volume (BS/BV), representing surface density, and ankle joint bone volume (BV), respectively. **(C) **Representative three-dimensional renditions of the knee joint scanned by micro computed tomography. The one-way ANOVA test with Tukey's post hoc comparison was used for statistical evaluation of all parameters shown in the bar plots. #*P *< 0.05 for the difference versus collagen-induced arthritis (CIA) + PBS.

Similar results were found in the periarticular bone in the knee joints where the BS/BV was increased compared to age-matched control mice, although the difference did not reach statistical significance (Figure 4A). This increase was not observed in the Scl-AbI-treated group and again this appeared to be due to an increase in bone volume (Figure 4B) from the endosteal surfaces. Qualitative analysis of the three-dimensional renditions of the knee joints (Figure 4C) confirmed that Scl-AbI treatment had been unable to prevent the external focal erosion on the periarticular surfaces.

### Therapeutic study - treatment with Scl-AbI has no effect on clinical scores

**Figure 5:**
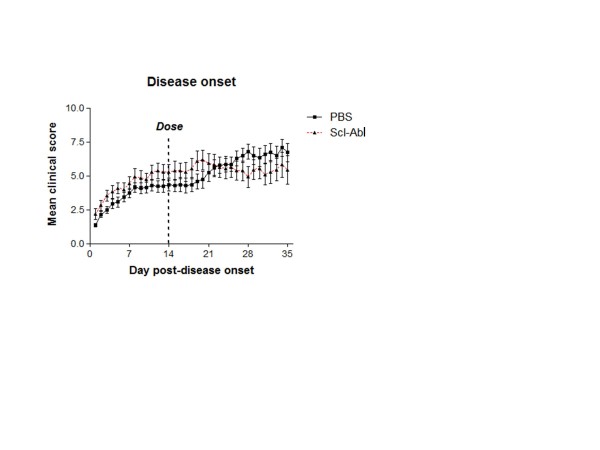
**Therapeutic administration of sclerostin antibody to arthritic mice has no effect on clinical scores**. Two groups of male DBA/1 mice were sensitised to collagen type II to induce arthritis. Starting from 14 days post onset of arthritis (that is the disease progression was synchronised in all mice before starting the treatment) mice were treated subcutaneously once a week with either vehicle (collagen-induced arthritis (CIA) + PBS; *n *= 14) or 10 mg/kg sclerostin antibody (Scl-AbI) (CIA + Scl-AbI; *n *= 11). The therapeutic study was terminated 21 days after starting treatment. Clinical score of arthritis was evaluated daily and expressed as the mean ± standard error of the mean in each group. Differences between clinical score were analysed by Mann-Whitney test.

Fifteen mice per group were sensitised to CII. Out of these, 14 mice developed disease in the PBS assigned group and 11 developed disease in the Scl-AbI assigned group, which indicates a robust disease induction before dosing was initiated. PBS and Scl-AbI were only administered to mice with active disease. Scl-AbI-treatment from 14 days post disease onset had no statistically significant effect on the mean clinical score throughout the course of the therapeutic study (Figure 5). As in the prophylactic dosing study, the body weight of the mice (approximately 23 g) in both the control and SclAbI-dosed groups varied little during the course of the experiment.

### Therapeutic study - treatment with Scl-AbI fully reverses whole body BMD, axial and tibial trabecular bone losses in CIA mice

**Figure 6:**
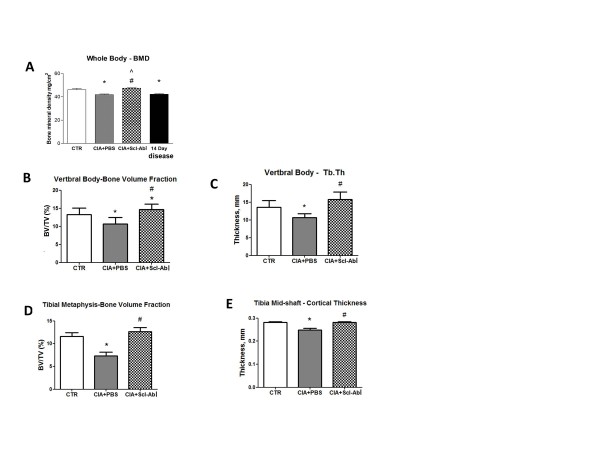
**Therapeutic administration of sclerostin antibody to arthritic mice reverses systemic bone loss**. **(A) **Bar plot represents mean (and standard deviation of) bone mineral density (BMD) assessed by dual X-ray absorptiometry (DEXA) in vehicle (collagen-induced arthritis (CIA) + PBS; *n *= 14) or sclerostin antibody (Scl-AbI) (CIA + Scl-AbI; *n *= 11) treated mice. An additional group of untreated diseased mice (*n *= 7) were scanned 14 days after onset of arthritis. These mice were used to represent baseline BMD at initiation of therapeutic dosing. BMD in age-matched healthy control mice (CTR; *n *= 4) was also assessed. **(B, C) **Bar plots represent mean (and standard deviation of) trabecular bone volume fraction (BV/TV) in the L5 vertebral body and trabecular thickness (Tb.Th). **(D, E) **Bar plots represent mean (and standard deviation of) trabecular bone volume fraction (BV/TV) the tibial metaphysis and cortical thickness of the cortical bone in the tibia midshaft. The one-way ANOVA test with Tukey's post hoc comparison was used for statistical evaluation of all parameters shown in the bar plots. *P < 0.05 for the difference versus control (CTR); #*P *< 0.05 for the difference versus CIA + PBS.

Mice scanned 14 days after the onset of arthritis confirmed that whole body BMD loss was already established at the start of the treatment and also revealed that disease progression did not further decrease BMD (Figure 6A). Arthritic mice treated with PBS showed a statistically significant decrease in whole body BMD as compared to age-matched control mice 35 days post disease onset (Figure 6A). Treatment with Scl-AbI restored whole body BMD to the same level as the healthy untreated control mice (Figure 6A, E).

Arthritic mice treated with PBS showed a statistically significant decrease in the bone volume fraction (BV/TV) in L5 vertebral body (Figure 6B), which was primarily due to thinning of the trabeculae (Figure 6C), consistent with findings in the prophylactic study. Therapeutic treatment of the arthritic mice with Scl-AbI significantly increased BV/TV and trabecular thickness compared with PBS treated and with BV/TV even significantly higher than in untreated healthy controls (Figure 6B). As in the prophylactic study, the trabecular BV/TV at the tibial metaphysis was significantly decreased by CIA but recovered by Scl-AbI treatment to the same level as the untreated healthy controls (Figure 6D). The mid-diaphyseal cortical bone thickness was also significantly reduced in arthritic mice treated with PBS compared to healthy untreated mice. Arthritic mice treated with Scl-AbI achieved an average tibial cortical thickness similar to the healthy untreated mice (Figure 6E).

### Therapeutic study - treatment with Scl-AbI does not repair focal bone erosions in CIA mice

**Figure 7:**
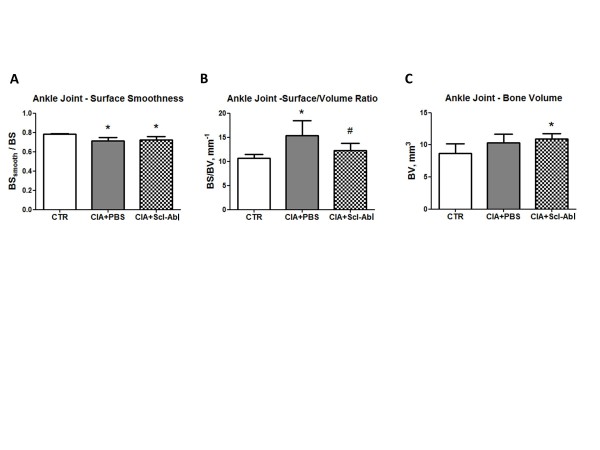
**Therapeutic administration of sclerostin antibody to arthritic mice does not repair focal bone erosion in periarticular bone in the ankle joint**. **(A, B, C) **Bar plots represent mean (and standard deviation of) smoothness parameter (BSsmooth/BS), ratio between bone surface and bone volume (BS/BV), representing surface density, and ankle joint bone volume (BV), respectively. CTR, age-matched healthy controls (*n *= 4). The one-way ANOVA test with Tukey's post hoc comparison was used for statistical evaluation of all parameters shown in the bar plots. *P < 0.05 for the difference versus control (CTR); #*P *< 0.05 for the difference versus collagen-induced arthritis (CIA) + PBS.

The periarticular bone in the ankle joints of arthritic mice showed a statistically significant decrease in the parameter evaluating surface smoothness (BSsmooth/BS, Figure 7A) and increase in the parameter quantifying surface density (BS/BV, Figure 7B) compared to age-matched control mice. Treatment of arthritic mice with Scl-AbI was able to abrogate the increase in the BS/BV (not statistically different from healthy untreated controls) but not the decrease in BSsmooth/BS. This effect appeared to be due to a significant increase in bone volume (Figure 7C), rather than a reduction of the superficial pitting as for the prophylactic study. This was confirmed by the qualitative analysis of three-dimensional renditions of the ankle joints of the CIA mice of the therapeutic study (data not shown as very similar to the images shown for the prophylactic study).

## Discussion

In this study, we showed that an antibody to sclerostin (Scl-AbI) can prevent or restore inflammation-induced systemic bone loss in a murine model of rheumatoid arthritis and had no significant effect on inflammation as assessed by the clinical score (paw inflammation, Figure 1B and Figure 5A) or on the disease incidence (in the prophylactic study, Figure 1A). The systemic decrease in BMD seen in patients with RA is well established in epidemiologic studies and can increase fracture risk especially in the vertebrae [24]. The effects of RA on systemic bone loss in patients were replicated in our murine model as shown by a decrease in whole body BMD (Figure 2G). Indeed by subdividing the sensitised mice into diseased and non-diseased (Figure 2G), we have shown that systemic bone loss depends on the onset of the local inflammation in the appendicular joints. Several studies have shown that strategies aimed at reducing inflammation (such as TNF-alpha blockade) are effective in reducing bone loss but not in rebuilding lost bone [24]. Treatment with Scl-AbI not only preserved whole body BMD in diseased animals but also increased it to levels above that in healthy control mice (Figure 2G). The same trend was found in a microCT analysis of the L5 vertebra (Figure 2A, B, C). Surprisingly, the trabecular bone in the tibial metaphysis followed the same trend, despite its proximity to the areas of focal erosion (Figure 2D, E, F). Treatment with Scl-AbI was also effective in restoring whole body BMD and microstructural parameters in the therapeutic study (Figure 6).

In our previous study on chronic inflammatory bone loss in a mouse IBD model [18], we found that the increase in whole body BMD, and bone microstructural parameters induced by Scl-AbI treatment, was associated with a reduction of inflammation-increased TRAPC5b and a restoration of inflammation-depressed osteocalcin serum levels, which corresponds to increasing bone formation and decreasing bone resorption. It seems likely that similar mechanisms operated to maintain/restore whole body BMD in arthritic animals treated with Scl-AbI. However, in the arthritis model, these mechanisms were not effective at modulating focal erosion in the periarticular bone of the joints affected by the disease. This was clearly demonstrated using two independent structural parameters for assessing bone focal erosion, namely BS/BV (which depends on changes in the bone volume) and BSsmooth/BS (independent of changes in bone volume). These parameters revealed that the blockade of sclerostin mainly stimulated bone mass accrual on the endosteal surface but not on the periosteal surface of the arthritic periarticular bone (Figure 3, Figure 4 and Figure 7). It is not clear why Scl-AbI is unable to prevent focal bone loss but it is possible that the intensity or nature of the inflammatory insult in the immediate vicinity of the joint may be different from the insult at more distant sites. The ability of Scl-AbI to mediate endosteal but not periosteal bone formation in the inflamed joints might also be attributed to a change in the inflammatory insult at the two sites. Diarra and co-investigators reported that an antibody to DKK-1 prevented focal bone loss in a different mouse model of RA. Dkk-1 and Scl are both regulators of the Wnt signalling pathway (although there seem to be differences in the members of the Wnt pathway that they inhibit) [16]. However, it is difficult to assess if the transgenic TNF model used by Diarra *et al*. generates the same local inflammatory environment as that observed in the CIA model reported here. Interestingly, another difference has been reported between Scl-AbI blockade and blockade of DKK-1. Whilst DKK-1 blockade appeared to promote the formation of osteophytes [17], sclerostin blockade was not associated with osteophyte formation [25]. Interestingly, a recent parallel publication [25], has shown that that Scl-AbI was able to also protect periarticular bone erosion in the same transgenic TNF model of RA used by Diarra and colleagues. This suggests that either the two models (CIA versus TNF transgenic) lead to very different degrees of joint inflammation or that other inflammatory cytokines, besides TNF-alpha, are present in the arthritic joints of our CIA model.

Further immune-localisation studies are required to assess the distribution of sclerostin in respect to key inflammatory cytokines (for example TNF-alpha, IL-1, IL-6, IL-17 and so on) along the periosteal and endosteal surfaces of the arthritic periarticular bone before firm conclusions can be drawn about the function of sclerostin in focal erosion areas.

In agreement with our previous study on IBD-induced bone loss [18], Scl-AbI had no effect on systemic inflammation, measured by circulating IgGs (Figure 1C, D, E). This is consistent with other studies where bone-specific treatments have failed to modulate immune reactions [15,26-29].

## Conclusions

In a murine model of rheumatoid arthritis, treatment for 21 days with an antibody to sclerostin reversed a decline in several axial and appendicular bone microstructural properties associated with chronic inflammation. However, treatment with Scl-AbI did not affect markers of disease severity or prevent/reverse the development of focal bone erosions in the proximity of inflamed joints. Thus treatment with an antibody to sclerostin may be useful in reducing fracture risk in patients suffering from long-term systemic inflammatory disease but used alone it may not prevent local joint destruction.

## Abbreviations

BMD: bone mineral density; BS: bone surface; BSA: bovine serum albumin; BSsmooth: computationally smoothed bone surfaces; BV: bone volume; CFA: complete Freund's adjuvant; CIA: collagen-induced arthritis; CII: chicken type II collagen; DEXA: dual-energy X-ray absorptiometry; DKK-1 and DKK-2: Dickkopf-1 and -2; ELISA: enzyme-linked immunosorbent assay; FLSs: fibroblast-like synoviocytes; IBD: inflammatory bowel disease; IL-1b, IL-6 and IL-15: interleukin-1beta, -6 and -15; MMP-3: matrix metalloproteinase-3; microCT: micro computed tomography; OPG: osteoprotegerin; PBS: phosphate-buffered saline; RA: rheumatoid arthritis; RANKL: nuclear factor-kappaB ligand; Scl: sclerostin; Scl-AbI: sclerostin antibody; sFRP1 and sFRP2: soluble frizzled receptor protein -1 and -2; TNF-alpha: tumor necrosis factor alpha; Wnt-1, Wnt-7b, Wnt10b and Wnt-5a: wingless-type MMTV (mouse mammary tumour virus) integration site family, member 1, 7b, 10b and 5a.

## Competing interests

AV, AM and MR are employed by UCB Pharma. MM was a former employee at UCB Pharma and is currently in a joint academic position 50% Imperial College London and 50% Oxford University. MR owns stock options in UCB Pharma.

## Authors' contributions

MM and AV are responsible for study design, data acquisition and analysis, and drafting and revising the manuscript. AM and MR are responsible for study design, data analysis and interpretation, and revision and final approval of the manuscript. All authors have read and approved the manuscript for publication.
